# Epidemiologic Studies of Isoflavones & Mammographic Density

**DOI:** 10.3390/nu2010035

**Published:** 2010-01-19

**Authors:** Gertraud Maskarinec, Martijn Verheus, Jeffrey A. Tice

**Affiliations:** 1 Cancer Research Center of Hawaii, 1236 Lauhala Street, Honolulu, HI 96813, USA; Email: m.verheus@nki.nl; 2 Department of Medicine, University of California, San Francisco, 1701 Divisadero Street, San Francisco, CA 94143, USA; Email: jeff.tice@ucsf.edu

**Keywords:** breast cancer risk, mammographic density, soy foods, isoflavones, Asian ethnicity, epidemiology, randomized trials

## Abstract

Isoflavones, phytoestrogens in soy beans with estrogen-like properties, have been examined for their cancer protective effects. Mammographic density is a strong predictor of breast cancer. This review summarizes studies that have examined the association between isoflavones and breast density. Observational investigations in Hawaii and Singapore suggest slightly lower breast density among women of Asian descent with regular soy intake, but two larger studies from Japan and Singapore did not observe a protective effect. The findings from seven randomized trials with primarily Caucasian women indicate that soy or isoflavones do not modify mammographic density. Soy foods and isoflavone supplements within a nutritional range do not appear to modify breast cancer risk as assessed by mammographic density.

## 1. Introduction

The low breast cancer rates in Japan as compared to Western countries and epidemiologic evidence [1,2] offer support for the hypothesis that soy intake protects against breast cancer. Investigations among women with Japanese and Chinese ancestry suggest that beneficial effects are more likely to be present in women who consumed soy during childhood and adolescence [2,3,4]. This review summarizes investigations that have examined the association between isoflavones and mammographic density as a marker for breast cancer risk. In addition, we present background information on the potential mechanisms of action for isoflavones and on the concept of mammographic density as a surrogate outcome for breast cancer and its relation to steroid hormones.

## 2. Background

### 2.1. Isoflavones

Soy and soy derived foods are by far the richest source of isoflavones (genistein, daidzein, and glycitein) in the human diet [5]. Isoflavones are considered phytoestrogens because of their estrogen-like structure and properties [6]. Daily isoflavone intake has been estimated as 25-50 mg in Asian countries, where traditional soy foods, such as tofu and soy milk, are commonly consumed [7] but is less than 1 mg in most Western countries [1,2]. Red clover is another source of isoflavones; biochanin A and formononetin yield genistein and daidzein after conversion in the intestinal tract [8]. Due to their estrogen-like structure, isoflavones have the ability to bind to estrogen receptors (ER). Their preference for ERβ over ERα has been proposed as the basis for their protective action against cancer [9]. ERα expression appears to be positively correlated with cell proliferation in mammary tumors in soy-treated rats [10], whereas ERβ transfection studies have demonstrated diminished proliferation and invasion in cell lines [11] suggesting that the relative proportion of the two receptors is important [12]. The frequency of ER expression may also contribute to the lower breast cancer risk among Japanese as compared to Caucasian women [13]. In the absence of steroidal estrogens, isoflavones appear to have weak estrogenic effects, whereas in the presence of steroidal estrogen, they exert an antagonistic effect through a competitive, ER-mediated mechanism [14]. Thus, isoflavones may prevent the proliferative effect of sex steroids on breast tissue, thereby lowering breast cancer risk. 

If isoflavones act on breast cancer risk through an antiestrogenic mechanism, it is possible that isoflavone supplementation in premenopausal women with high circulating estrogen levels is not sufficient to influence breast cancer risk. However, in postmenopausal women with low circulating estrogen levels primarily derived through conversion from androgens in adipose tissue, isoflavones from foods or supplements may be more likely to exert an estrogen-like action on the breast raising some concern about adverse effects [15]. In addition, several other mechanisms of action have been proposed as mediators between soy intake and breast cancer risk. Isoflavones may lower estrogen levels through stimulation or upregulation of sex hormone binding globulin in the liver [16], downregulation of enzymes involved in estrogen biosynthesis such as aromatase that converts androgens to estrogens [17], inhibition of 17,3-hydroxysteroid-oxido reductase that converts estrone to the more potent estradiol [18], and effects on the intestinal flora that may decrease reabsorption of estradiol [19,20]. In addition, evidence has emerged that soy may protect against breast cancer through non-estrogenic pathways such as antioxidative, anti-inflammatory effects, or inhibition of topoisomerase or tyrosine kinase [21,22]. Finally, the weak estrogenic effects of isoflavones early in life [23] may achieve or accelerate differentiation of breast tissue structures similar to an early pregnancy and, thereby, make the tissue less susceptible to carcinogens [24,25]. 

### 2.2. Mammographic Density and Breast Cancer Risk

Mammographic density, one of the strongest breast cancer risk factors, describes the radiographic appearance of the female breast [26]. Fat, which is radiolucent, appears dark on mammograms. Epithelial and stromal tissues, on the other hand, appear white or radiodense and are collectively described as mammographic density. Women in the highest density category have a 4-6 fold higher breast cancer risk than those with very low densities [26]. Qualitative and quantitative methods have been used for density assessment on digitized or digital mammographic images. The first density assessment method was presented by Wolfe [27]. This qualitative approach classified the appearance of breast images into four categories and was later expanded to five classes by Tabar [28]. Later, visual estimation techniques provided quantitative results as a continuous outcome [29,30]. Computer-based methods using scanned mammographic images became widely used in the 1990s, also measure density on a continuous scale, and simplified the assessment of large numbers of films [31,32]. The most commonly used program (Cumulus) was developed in Toronto (Canada) [31,33]. When the different assessments methods are compared, they all describe the associations with well known breast cancer risk factors but the quantitative methods appear to capture more information than the qualitative methods [34]. Several volumetric approaches have been developed, although not commonly used yet, and have the goal to capture three- instead of two-dimensional images [35,36].

### 2.3. Steroid Hormones and Mammographic Density

Hormone replacement therapy (HRT), in particular containing progestin, has been shown to increase mammographic densities [37]. As observed in several trials, breast density increased between 3-5% in women taking estrogen/progestin combination therapy over at least one year, while no change was observed for the placebo and the estrogen only groups [38,39]. Such effects were expected based on the epidemiology of breast cancer and the relation of ovarian hormone levels to breast cell proliferation [40] and were confirmed by the Women's Health Initiative showing that combined estrogen-progestogen therapy significantly increases the risk of breast cancer [41]. A dramatic reduction of mammographic density was also observed in trials with a gonadotropin-releasing hormone agonist [42] and with tamoxifen [43] but not raloxifene [44]. Although these investigations support the use of breast density as a marker for breast cancer risk [40], it remains unclear, if the mammographic changes are causally linked to the higher breast cancer risk or if two separate pathways are involved [37,45].

In comparison to HRT, the relation between mammographic density and endogenous sex steroids is less clear [45]. Whereas one cross-sectional investigation observed a significant association with estrogens [46], other reports did not [47,48,49]. Adjustment for body mass index (BMI) generally weakened the association but residual confounding for adiposity cannot be excluded [45]. It appears likely that mammographic density and circulating sex steroids are strongly and independently associated with breast cancer risk and that breast density does not mediate the effect of the endogenous hormones [48]. Similar to HRT, circulating progesterone may influence breast density more than estrogens. Among premenopausal women, progesterone showed a stronger association with mammographic density than estrogen [50].

## 3. Results

Our search for studies on isoflavones and breast density identified six reports (Table 1) from five cross-sectional investigations [51,52,53,54,55,56], one longitudinal analysis [57], and eight publications from seven randomized trials (Table 2). We are summarizing findings for observational studies and randomized trials separately. A final section reviews publications that have examined equol-producing status and breast density. With one exception [52], all observational studies included women of Asian ancestry. On the other hand, all, but two interventions from Hawaii that included Japanese American women [58,59], were conducted in Caucasian populations who did not regularly consume foods with high isoflavone content.

### 3.1. Observational Studies

The cross-sectional studies (Table 1) and the longitudinal investigation examined the association between soy intake, as assessed by food frequency questionnaires, and breast density among healthy pre-and postmenopausal women [51,52,53,54,55,56,57]. A study in Hawaii detected a weak positive association between soy intake and percent mammographic densities [51]. While average breast area and the non-dense area were approximately 25% smaller among women in the highest soy intake category compared with women in the lowest category, percent densities were 11% greater with higher soy intake. The dense areas appeared to be slightly smaller with increasing soy intake but the trend test was not significant. After stratification by ethnicity, the findings indicated non-significant differences by ethnicity. Whereas the relation was inverse, although not statistically significant, among Chinese and Japanese women, Caucasians with a higher soy intake had slightly higher mammographic densities. A small German study with a very low mean isoflavone intake (around 0.1 mg per day) reported a non-significantly higher isoflavone intake among pre- and postmenopausal women with non-dense breasts [52]. When breast density among 601 Japanese women was evaluated in relation to dietary soy isoflavones, no significant associations of isoflavones with percent density in pre- and postmenopausal women were detected [55]. In fact, breast density was non-significantly higher for women in the highest quartile, similar as for Caucasians in the Hawaii study [51]. The Japanese study used an automated mammographic mass detection method different from the approach applied in the other cross-sectional reports [55].

Two reports from the same study population in Singapore indicated possible beneficial effects of soy on breast density [53,54]. Dietary and other lifestyle profiles were compared between subjects classified as displaying high risk and low risk parenchymal patterns [53]. After adjustment for energy intake and other potential confounders, dietary soy protein intake was inversely related to the risk of high-risk parenchymal pattern; an estimated risk of 0.41 was observed when comparing extreme quartiles [53]. When a quantitative method of density assessment was applied to the same mammographic images, higher soy intake was associated with 4-5% lower percent densities [54]. A larger investigation of 3,315 Chinese women, also in Singapore, examined the combined effect of soy foods and green tea on breast density [56]. While the difference between the highest quartile and the lowest three quartiles combined was less than 2%, it was statistically significant in post- but not in premenopausal women. After adjustment for green tea intake, the association between soy and density was no longer significant indicating a stronger effect of green tea than soy on breast density [56]. 

**Table 1:** Cross-sectional studies on soy/isoflavones and mammographic densities.

Author, Year	N Status	Isoflavone intake (mg)		Assessment method3	Results for lowest and highest intake categories	
Maskarinec, 2001 [51]	514 Pre & post	Asians	8^1^	Computer-assisted	Asians	38.2% and 34.5%; p = 0.31
		Whites	4^1^		Whites	26.7% and 30.7%; p = 0.06
Nagel, 2005 [52]	54 Pre & post	0.1		Wolfe categories	0.01 *vs.* 0.19 mg isoflavones in women with dense *vs.* non-dense breast; p = 0.26	
Nagata, 2005 [55]	601 Pre & post	Pre	42	Automated	Pre	30.2% and 37.8%; p = 0.28
		Post	75		Post	9.7% and 13.1%; p = 0.33
Jakes, 2002 [53]^2^	406 Pre & post	14		Tabar categories	Odds ratio of 1 and 0.44 to have dense breasts; p = 0.07	
Ursin, 2006 [54]^2^	380 Pre & post	15		Computer-assisted	26.1% and 21.2%; p = 0.03	
Wu, 2008 [56]	3,315 Pre & post	Pre	17	Computer-assisted	25.7% and 26.3%; p = 0.28	
		Post	18			

Notes: ^1^ Estimated isoflavones in 38 and 22 g soy foods; ^2 ^Same study population; ^3 ^All methods are quantitative except for Tabar and Wolfe categories

Despite three suggestive reports [51,53,54], the negative results in the two larger cross-sectional investigations [55,56] indicate that the effect of regular soy intake on mammographic density, if it exists, is weak. Additional observational evidence comes from a nested case-control study conducted in Hawaii [57]. Early life soy intake as assessed by a lifetime soy questionnaire was weakly related to lower percent densities, while soy consumption during adulthood predicted significantly higher densities. The rate of change in breast density over time was not significantly affected by soy intake although women who consumed soy as adults appeared to experience a faster decline in density than women who consumed soy as early in life.

### 3.2. Randomized Trials

Of the eight reports describing seven randomized trials (Table 2), hormonal outcomes were the primary endpoints for three studies among premenopausal women [58,59,60], bone density for two trials in postmenopausal women [61,62], and a combination of health effects to assess safety of isoflavones for the two other interventions [59,63]. The hypothesis for clinical trials with the goal to examine breast density was that treatment with soy foods or isoflavones would decrease mammographic density as a marker of breast cancer risk. Of the seven randomized trials, two included only premenopausal women [58,59], three only postmenopausal subjects [61,62,63], and two pre- and postmenopausal women [60,64]. Soy foods, i.e., tofu, soy milk, soy nuts, and soy bars, were used in one [59], soy protein powder in one [63], soy-based isoflavone supplements in two [58,62], a genistein only supplement in one [61], and isoflavone tablets from red clover, primarily biochanin A and formononetin, in two interventions [60,64]. The duration of the trials ranged between 1-3 years, while the sample sizes varied from 30-401 participants. All interventions randomized healthy women, although at high breast cancer risk in one trial [64] and with high breast density in another one [60]. 

**Table 2:** Randomized trials with soy/isoflavones and breast density as outcome.

**Author Year**	**N Status**	**Treatment**	**Iso dose (mg)**	**Duration (years)**	**Assessment method4**	**Change in density (%)**			
						**Controls**		**Intervention**	
Maskarinec 2003 [58]	30 Pre	Isoflavone supplement	100	1	Computer-assisted	0.4		2.5	
Maskarinec 2004 [59]	213 Pre	Soy foods	50	2	Computer-assisted	-4.1		-2.8	
Powles 2008 [64]	401 Pre & post	Red clover supplement	40	3	Visual estimation	Pre	-3.0	Pre	-6.6
						Post	-8.0	Post	-6.9
Verheus 2008 [63]	126 Post	Soy powder	99	1	Computer-assisted	-4.6		-2.5	
Marini 2008 [61]	138 Post	Genistein supplement	54	3	Wolfe categories	13.4 ^1^		22.5 ^1^	
					Computer-assisted	-0.4 ^2^		-0.4 ^2^	
Atkinson 2004 [60]^3^	177 Pre & post	Red clover supplement	45	1	Visual estimation	-3.9		-3.2	
Kataoka 2008 [65]^3^	177 Pre & post	Red clover supplement	45	1	Computer-assisted	-0.5		-2.1	
					Volumetric	0.8		0.3	
Maskarinec 2009 [62]	358 Post	Isoflavone supplement	80 & 120	2	Computer-assisted	-1.4/y		80 mg	-1.6/y
								120 mg	-1.3/y

Notes: ^1^% improved category; ^2^Arbitrary units, not percent density; ^3^Same study population; ^4^All methods are quantitative except for Wolfe categories

In most studies, the mammograms had been obtained as part of regular medical care; the subjects were not exposed to additional radiation as part of the interventions. Therefore, due to current screening recommendations, women under 40 years could not be enrolled into the interventions. All trials except the soy-food based trial [59] were blinded and administered a placebo pill [58,60,61,62,64] or a milk-based powder [63] to subjects in the control group. In comparison to regular soy intake in Asian countries [7], the administered doses were substantially higher (80-120 mg) in three trials [58,62,63], while they were in the high nutritional range in the others [59,60,61,64]. Compliance was addressed by different methods in each trial and appeared satisfactory; overall drop-out rates were within the acceptable range. No major adverse effects were reported by any of the studies. The most recent trial was the multicenter Osteoporosis Prevention Using Soy (OPUS) study with a primary endpoint of bone density [62]. In a double-blinded and placebo-controlled design, two relatively high doses of 80 or 120 mg/d of isoflavones were administered for two years. 

All but three reports [60,61,64] used the same computer-assisted Cumulus method [31]. One investigation used both the Wolfe scale and a digitized quantification and detected no significant change with either method [61]. Another report compared the outcomes using three different assessment methods [65]. Re-examining the same mammograms as in the earlier report with Wolfe patterns [60], the investigators applied Cumulus and the volumetric standard mammogram form (SMF) method. Whereas no significant treatment effect was observed with the visual estimation and the SMF method, the intervention group showed somewhat greater reduction of percent density by Cumulus in comparison to controls (P = 0.04) but the difference was small (−2.1 *vs.* −0.5%) and was attenuated after adjustment for baseline percent density (P = 0.06). The OPUS trial had the challenge that some of the mammograms were digital, whereas the majority were regular film mammograms [62]. Fortunately, 83% of women received the same type of mammography throughout the study. 

In the 2-year nutritional trial, the effects of lifetime soy intake on mammographic density were also explored [59]. Soy intake since age 20 as measured by the lifetime soy questionnaire was negatively associated with annual change in percent density (P = 0.007). Women who reported at least one serving per week during adulthood experienced a greater decline in densities than women with lower intakes indicating that soy consumption throughout life may be associated with breast density, although this could be confounded by BMI and other lifestyle factors associated with high lifetime soy consumption.

The eight reports, including the three high-dose trials, were in agreement that isoflavone or soy treatment did not result in statistically significant changes in mammographic densities when compared to the control arm [58,59,60,61,62,63,64,65]. However, with one exception [58], breast density decreased during the duration of the trial due to aging of the breast tissue as observed in longitudinal mammographic density studies [57,66]. For example, in the OPUS study mean percent density decreased by 1.6% per year across groups [62] and in the soy food trial annual declines of 1.2% and 1.9% were observed among intervention and control subjects [59].

### 3.3. Equol-Producer Status and Breast Density

Soy consumption may have a stronger association with mammographic density in subjects who are able to metabolize the isoflavone daidzein to equol than those who cannot [67]. Several mechanisms of action have been proposed [68]. Equol may bind to ER with greater affinity than other isoflavonoids, the equol producing phenotype may be associated with other factors influencing breast density, or differences in intestinal bacterial populations may result in different reproductive hormone metabolite profiles and circulating hormone concentrations. Three studies assessed equol excretor status after a challenge with soy protein. The equol producers among a group of sedentary postmenopausal women in Seattle were found to have lower mammographic densities than the non-producers [68]. These results suggest that particular intestinal bacterial profiles that facilitate isoflavone metabolism may be associated with lower postmenopausal mammographic density. However, two other studies that stratified by equol excretor status did not detect any differences in breast density [63,69]. In a Dutch trial, equol producer status was assessed at the end of the trial using a 3-day soy challenge but the results were not affected by equol-producer status [63]. No significant independent associations of equol status or soy intake with percent density although a significant interaction between these factors was observed in another cross-sectional soy-challenge investigation [69].

## 4. Discussion and Conclusions

Although cross-sectional investigations in Hawaii and Singapore suggest slightly lower breast density among women of Asian descent with regular soy intake [51,53,54], the two larger studies with Japanese and Chinese women did not observe any conclusive evidence for a protective effect [55,56]. With great consistency, the randomized trials indicate that soy or isoflavones do not modify mammographic densities among adult pre- and postmenopausal Caucasian women. Whereas the relatively short durations and the limited sample sizes may have been responsible for a lack of an effect on breast density in the trials presented here, it appears probable that isoflavones do not influence breast density in adult women who grew up in Western countries and consumed few isoflavones in their regular diet. A lack of an effect on mammographic density would not rule out a protective effect of isoflavones against breast cancer as indicated by case-control studies [1,2]. A modification in breast cancer risk through an intervention may not necessarily be reflected in breast density. Soy isoflavones may affect breast cancer through a different pathway than mammographic densities in the same way as proposed for endogenous steroid hormones [70,71] and postmenopausal obesity [72]. 

The body of literature discussed here has a number of limitations. Nearly every trial reviewed here administered a different isoflavone containing product (Table 2) or even used only one aglycone isoflavone [61]. Non-fermented soy foods typically contain glucosides that are hydrolyzed to aglycones by intestinal bacteria and isoflavone aglycones may be absorbed faster and in greater amounts than glucosides [73]. The use of red clover as a source of isoflavones may have also affected the results given that biochanin A and formononetin are structurally different from soy isoflavones [8,60,64]. As shown for LDL cholesterol reduction, soy protein may be more effective than isoflavone supplements [74]. Despite the advantages of soy foods in that they replicate soy exposure in Asian countries, the one study using soy foods warrants cautious interpretation due to the lack of blinding [59]. The relatively high isoflavone doses used among women with low previous exposure may not be comparable to Asian soy exposures since early childhood [62,63]. Unfortunately, no data on the effects of soy in women under 40 years are currently available because the risk of radiation would outweigh the benefits of mammography. Therefore, it has not been possible to examine the hypothesis that soy exposure early in life reduces breast cancer risk to a greater degree than during adulthood [3,4]. 

Methodological problems in density assessment raise some questions about the validity of the findings. As shown in a comparative report [65], there was a suggestion of a greater reduction of percent density in the isoflavone group than controls, but only when breast density was measured by Cumulus. Thus, it is possible that slightly different conclusions could be drawn by using different methods to assess breast density. As in all cross-sectional studies, confounding by other nutritional or lifestyle factors, e.g., fruit and vegetable intake, BMI, or physical activity, may have biased the results. 

On the positive side, the lack of an isoflavone treatment effect on breast density offers some reassurance to those who are concerned about the possible risks of soy foods [15]. Concerns were raised that the estrogen-like effects of isoflavones may induce breast cell proliferation [75,76]. The evidence from the randomized trials reviewed here indicates that consuming soy foods within a nutritional range does not increase breast density as reported for HRT [37] and it remains possible that long-term soy exposure may offer some protection against breast cancer [53,54,57]. This idea is supported by a recent report from China that observed a lower risk of breast cancer death and recurrence among women with higher soy consumption [77]. Despite methodological challenges, we need to understand better in the future how soy affects breast cancer risk earlier in life. As shown in several studies among Asian women, soy exposure during adolescence may play a more important role in breast cancer prevention than during adulthood [3,4]. Novel breast imaging approaches, such as MRI [78], ultrasound [79], and DXA [80], will be needed to understand nutritional influences on breast density in adolescents and young women.
